# Concentration of Microparticles/Cells Based on an Ultra-Fast Centrifuge Virtual Tunnel Driven by a Novel Lamb Wave Resonator Array

**DOI:** 10.3390/bios14060280

**Published:** 2024-05-29

**Authors:** Wei Wei, Zhaoxun Wang, Bingnan Wang, Wei Pang, Qingrui Yang, Xuexin Duan

**Affiliations:** State Key Laboratory of Precision Measuring Technology & Instruments, Tianjin University, Tianjin 300072, China; wei_wei@tju.edu.cn (W.W.); wangzhaoxun@tju.edu.cn (Z.W.); wangbingnan@tju.edu.cn (B.W.); weipang@tju.edu.cn (W.P.)

**Keywords:** concentration, ultra-fast centrifuge, Lamb wave resonator array

## Abstract

The µTAS/LOC, a highly integrated microsystem, consolidates multiple bioanalytical functions within a single chip, enhancing efficiency and precision in bioanalysis and biomedical operations. Microfluidic centrifugation, a key component of LOC devices, enables rapid capture and enrichment of tiny objects in samples, improving sensitivity and accuracy of detection and diagnosis. However, microfluidic systems face challenges due to viscosity dominance and difficulty in vortex formation. Acoustic-based centrifugation, particularly those using surface acoustic waves (SAWs), have shown promise in applications such as particle concentration, separation, and droplet mixing. However, challenges include accurate droplet placement, energy loss from off-axis positioning, and limited energy transfer from low-frequency SAW resonators, restricting centrifugal speed and sample volume. In this work, we introduce a novel ring array composed of eight Lamb wave resonators (LWRs), forming an Ultra-Fast Centrifuge Tunnel (UFCT) in a microfluidic system. The UFCT eliminates secondary vortices, concentrating energy in the main vortex and maximizing acoustic-to-streaming energy conversion. It enables ultra-fast centrifugation with a larger liquid capacity (50 μL), reduced power usage (50 mW) that is one order of magnitude smaller than existing devices, and greater linear speed (62 mm/s), surpassing the limitations of prior methods. We demonstrate successful high-fold enrichment of 2 μm and 10 μm particles and explore the UFCT’s potential in tissue engineering by encapsulating cells in a hydrogel-based micro-organ with a ring structure, which is of great significance for building more complex manipulation platforms for particles and cells in a bio-compatible and contactless manner.

## 1. Introduction

The micro total analysis system (µTAS), also known as the Lab-on-a-Chip (LOC), is a highly integrated microsystem technology. It encapsulates functions such as sample pretreatment, execution of biochemical reactions, detection, and analysis within a single microchip, thereby efficiently completing a series of complex and precise bioanalysis and biomedical operations [1,2]. Among them, microfluidic centrifugation technology plays one of the core roles in LOC devices. Through centrifugal pumping, microvalves, micro-mixing, and separation of sample particles and fluids, it successfully constitutes indispensable sample preparation technology, as well as analysis and detection methods in the process of in vitro diagnosis and drug development [3]. Particularly in sample preprocessing, the rapid capture and enrichment of tiny objects (such as cells and particles) in the sample through microfluidic centrifugation helps to shorten the analysis cycle and greatly enhances the sensitivity and accuracy of biological detection and diagnosis [4,5,6]. However, due to the change in fluid characteristics under the microfluidic system, on the one hand, the viscosity effect dominates, weakening the effectiveness of the centrifugal separation mechanism. On the other hand, the low Reynolds number of the environment is not conducive to the natural formation of vortices.

Therefore, various passive and active technical strategies are developed to enhance vortex intensity to meet the needs of microfluidic centrifugation applications [7]. Passive microfluidic centrifugation technology primarily relies on the design of microchannels with specific geometric shapes, topological structures, or curvatures. This design induces vortices by utilizing the principles of inertial fluid mechanics [8,9]. Although these designs are simple and low-cost, maintaining effective fluid dynamics often requires the use of high-power pumps, which is not conducive to the miniaturization and overall integration of microfluidic control devices. In contrast, active methods depend on externally applied forces to drive the rotation within the system. These forces typically originate from electric fields [10,11], acoustic fields [12,13,14], and thermal fields [15]. Compared to passive methods, active methods have the advantage of real-time, precise control of vortex intensity, direction, and distribution. They are adaptable to complex, dynamic operational requirements and offer high flexibility [16,17].

The acoustic approach, as an active method in microfluidic centrifugation, offers a highly efficient and flexible solution to the challenge of vortex generation at the microfluidic scale [18]. It boasts several advantages, such as non-invasive biocompatibility, precise control through multi-parameter adjustments, strong penetration, compatibility with various microfluidic control materials, and highly localized generation of acoustic streaming [19,20,21,22,23]. Due to the transferability of vibrations, various waveguides [24] and acoustic black hole effects [25] can be combined to further generate acoustic fluid effects with multiple modes through wave modulation. Microfluidic centrifugation based on acoustic methods is mainly divided into two categories: indirect and direct methods. Indirect methods typically induce acoustic streaming by introducing a medium (microstructures or bubbles) that oscillates secondarily [26,27,28]. However, the reproducibility and robustness of streaming caused by indirect strategies are inferior to those of direct strategies. Direct strategies usually involve the direct coupling of sound waves into the fluid in the microfluidic system. In recent years, microfluidic centrifugation based on surface acoustic waves (SAWs) directly coupling into microfluidics has demonstrated the potential of centrifugation in applications such as particle concentration, separation, and micro-mixing. The pivotal objective lies in amplifying the net torque around the vertical axis to boost rotational speed and torque [29]. First, refining the electrode design and harnessing a focused SAW intensify the energy transferred into the fluid, thereby escalating the vortex velocity within the droplet [30,31,32]. Secondly, adjusting the distribution of the SAW, such as by introducing asymmetric or annular structures, can further enhance the streaming speed by adjusting the input power and the flow field distribution [29,33,34].

Despite their merits, these methodologies encounter several hurdles. The accurate placement of droplets poses a challenge, along with considerable energy loss due to off-axis positioning. Additionally, only a minute proportion of the input power imparted to the low-frequency SAW resonator is effectively conveyed into the fluid. Higher-frequency SAW fabrication is relatively difficult and hard to apply under high power. Therefore, it is challenging to increase the flow velocity and centrifuge speed, and the droplet capacity is typically below 10 μL [29]. For larger volumes, high streaming velocity is required. Based on higher frequency Lamb wave resonators (LWRs), rapid acoustic streaming energy conversion can be achieved, and it can be driven at a lower power [35].

In previous work, our research group has explored the acoustic streaming effects excited by a single LWR [36,37] and an array of four LWRs in a liquid environment, as well as using it for particle enrichment [35]. However, due to the presence of secondary vortices, it is challenging to form a single high-speed streaming tunnel for centrifuge applications. Therefore, in this work, we designed and manufactured a ring array composed of eight LWRs with novel structures. On one hand, this eliminates secondary vortices, allowing more energy to concentrate in the main vortex, maximizing the conversion efficiency from an acoustic wave to acoustic streaming. On the other hand, through the arrayed layout, an ultra-fast centrifuge tunnel (UFCT) is formed in the center of the microfluidic system in droplets with a larger fluid volume (50 μL). The UFCT allows the use of less power (50 mW) to achieve greater linear speed (62 mm/s), thereby achieving ultra-fast microfluidic centrifugation. Moreover, we have also explored the relationship between the fluid motion state and particle motion speed, particle size, and fluid viscosity and this impact on the UFCT, as well as the relationship with input power. We have triumphantly accomplished high-fold enrichment of 2 μm and 10 μm particles and have initially embarked on exploring the prospective utility of the UFCT in tissue engineering. Using hydrogel PEGDA as a scaffold, we encapsulated cells and formed a micro-organ with a ring structure, which contributes to the advancement of bio-compatible and non-invasive micro-assembly systems for particles and cells.

## 2. Materials and Methods

### 2.1. Composition and Working Principle of UFCT

**Figure 1:**
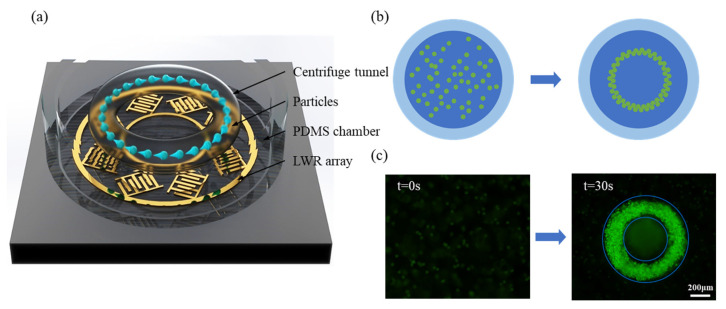
Working principle of UFCT for particle concentration. (**a**) Schematic diagram of UFCT for particle enrichment: the UFCT is driven by an LWR array, and the particle suspension is added to the PDMS ring as a sample reservoir. When LWR is turned on, the particles are rapidly concentrated into the central ring under the action of UFCT. (**b**,**c**) show the schematic diagram and experimental diagram of UFCT-concentrated particles after acting for 30 s and curing in hydrogel.

Figure 1a is a schematic diagram of the UFCT working principle, which is composed of a PDMS chamber, the sample, and a novel LWR ring array underneath. The main purpose of the novel LWR ring array design is to focus energy effectively into the liquid by utilizing its unique ring angle and reflection-grating capabilities, thereby improving the shape of the excited vortex, generating a UFCT in the central circular area, and adapting to the subsequent particles and cell-capture assembly. The working process is as shown in Figure 1b: a low-concentration sample (about 50 μL) is applied to the sample of the chamber. When the LWR array is turned on, the UFCT instantly forms in the entire field. Particles in the entire field rotate with the vortex, and due to the combined effect of the high-speed fluid shear force of the UFCT and the acoustofluidic effect, the particles in the entire field are dragged into the central vortex for capture and enrichment in about 30 s. As most particles are dragged into the vortex, the width of the ring trajectory significantly expands until the particles fill the entire virtual tunnel and no further enrichment can be carried out. After solidification in the hydrogel, the assembly can be taken out.

**Figure 2:**
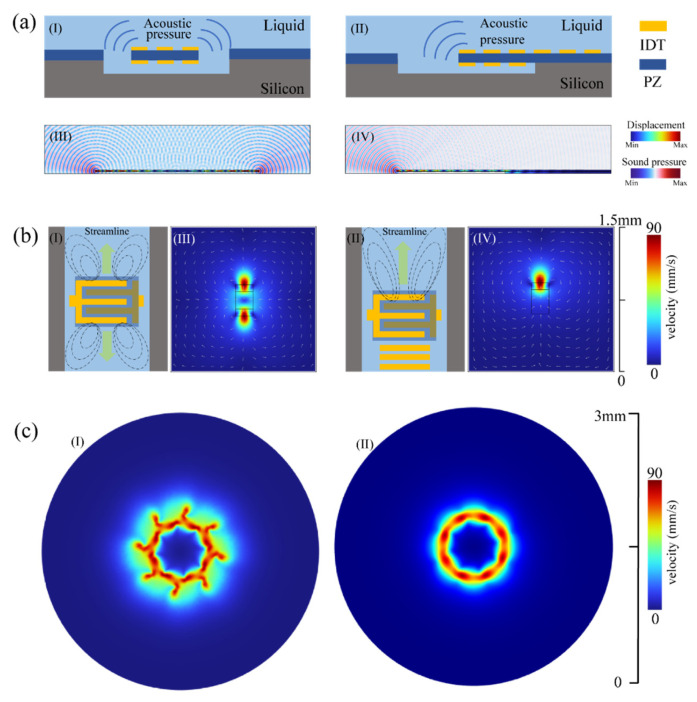
Principle design and simulation validation of traditional and novel LWRs: (**a**) Two-dimensional cross-sectional schematic and simulated displacement and acoustic pressure diagrams for a traditional LWR and a novel LWR. (**b**) Three-dimensional flow field propagation schematics and corresponding simulation diagrams for a traditional LWR and a novel LWR. (**c**) Schematic and simulated velocity distribution maps for traditional and novel LWR arrays.

In previous studies, the vortices behind the LWR made the flow field distribution complex [35]. To achieve a more uniform fluid flow in the chamber and facilitate the quick enrichment of particles to the predetermined annular orbit, this paper designed a novel LWR with a new structure. To verify its feasibility, namely whether it enhances the generation and velocity of the primary vortex and reduces the formation of secondary vortices, we conducted finite element analysis (FEA) comparisons between the new structure and the traditional structure. The fluid used for simulation was water, with a density of 997 $kg/m^{3}$ and a dynamic viscosity of 1 mPa·s. For all transient studies, the relative tolerance for convergence accuracy was set to 0.001. The traditional LWR has a suspended flat-edged structure as shown in Ⅰ of Figure 2a.

When the radio frequency signal is connected to the electrodes, an electric field is generated perpendicular to the plane of the thin film. The particles of the piezoelectric thin film vibrate on the plane of the film surface (ignoring the thickness, as the film is sufficiently thin). Due to the strong acoustic impedance difference at the free edge, a standing wave is formed in the thin film, resulting in resonance. The resonant frequency $f_{r}$ is determined by the spacing between the interdigital electrodes and the characteristics of the wave.

(1) $$f_{r}=c_{0}\sqrt{{\left(\frac{n}{2p}\right)}^{2}+{\left(\frac{m}{2L}\right)}^{2}}$$

Among them, n and m are both positive integers, p is the electrode spatial period, L is the electrode strip length, and $c_{0}$ is the wave velocity of Lamb waves in S0 mode. As shown in Ⅰ and Ⅲ of Figure 2a, when traditional LWRs operate in a liquid environment, the acoustic waves in the resonator propagate to the solid–liquid interface, where some of them leak into the liquid from both sides of the edge, resembling cylindrical waves. An optimized grating reflector is used to replace the air edges of the LWR so that the acoustic energy of the Lamb waves is directed from only one side of the resonator into the liquid, as indicated in Ⅱ and Ⅳ of Figure 2a.

To suppress standing wave leakage, periodic structure reflectors are employed, their efficiency influenced by factors such as strip unit count and inter-strip spacing. Grating spacing adheres to λ/4 and matches IDT widths for optimal reflection, and the theory links strip numbers positively to reflected wave energy [38,39]. For miniaturization and controlled reflection, just five oppositely placed molybdenum electrodes were used on each side. Further investigation was conducted into the velocity distribution of the novel LWR and traditional LWR in liquid at resonance frequencies. The designed device was coupled into the fluid for comparison. Given that cylindrical waves are generated only on one side of the electrode bars, we extracted the first-order velocity field from the linear acoustic domain, simplified it, and subsequently introduced it into Equation (2) to obtain the volume force:

(2) $$\left\langle F\right\rangle =-\left\langle \rho_{0}\left(\upsilon_{1}\cdot \nabla \right)\upsilon_{1}+\upsilon_{1}\nabla \cdot \rho_{0}\upsilon_{1}\right\rangle =-\rho_{0}\left\langle \left(\upsilon_{1}\cdot \nabla \right)\upsilon_{1}+\upsilon_{1}\nabla \cdot \upsilon_{1}\right\rangle$$

The acoustic vibration velocity field distribution $\upsilon_{1}$ is added to the domain where the volume force acts, with the fluid density denoted as $\rho_{0}$. The upper boundary of the fluid domain is set as an “open boundary” to simulate an infinitely large liquid environment. Specific simulation steps can be found in the Supplementary Materials. The steady laminar flow calculation results for the traditional and novel Lamb wave resonators are displayed in Figure 2b. Notably, the grating’s wave reflection significantly reduces leakage into the liquid, concentrating the acoustic pressure distribution on one side of the LWR.

As observed in Ⅰ and Ⅲ of Figure 2b, the strongest acoustic streaming effect occurs in the vicinity of the resonator device, diminishing with increasing distance from the device, consistent with the attenuation of acoustic waves in the fluid domain and the finite extent of the volume force. Under the influence of acoustic streaming, the fluid flows along the *x*-axis away from the device center towards both sides, followed by fluid movement along the *y*-axis towards the center, replenishing the displaced fluid. A circulation forms within the liquid, with a vortex present at the corners of the LWR. In contrast, as seen in Ⅱ and Ⅳ of Figure 2b, the adoption of an air-grating structure on one side renders the direction of acoustic wave leakage into the fluid unidirectional, generating volume force on only that side. Concurrently, the fluid intensity and gradient on the air-grating side have increased. Due to the unidirectionality of the volume force, the fluid moves along the *y*-axis in the direction of acoustic wave propagation, returning to the rear of the resonator, creating vortices on both sides. This configuration results in a more concentrated energy distribution and a reduction in the formation of secondary vortices.

Considering both the continuity of the flow field and the optimization of power consumption, we concluded that an array composed of eight devices represents the most suitable configuration (the specific design concept can be found in the Figure S1). We arranged the LWR array such that the top corners of the eight novel LWRs lay on a common circumference, with inner and outer electrode rings providing electrical connections. Each device was positioned at a 45° angle relative to its neighbors, resulting in a symmetrical distribution. In an open-flow field, a single LWR generates vortices approximately 400 μm from its top corner, while the radius of the array’s inner circle is about 200 μm. This configuration ensures overlapping regions of cylindrical wave generation near the inner side of each resonator, effectively rendering the volume force a continuous force in a circumferential direction.

As depicted in Figure 2c, we conducted a three-dimensional simulation of the complex composite field. At the array’s center, a primary vortex formed with the highest velocity, rotating counterclockwise. Comparing the flow fields generated by traditional and novel LWR arrays, in Ⅰ of Figure 2c, the traditional LWR array still exhibited velocity distributions of secondary vortices, with speeds amounting to 70% of the primary vortex. Conversely, in II of Figure 2c, the novel array collectively excited 16 vortices, all of which coalesced into the dominant primary vortex. The resulting primary vortex exhibited a more uniform velocity distribution, essentially eliminating the influence of secondary vortices.

### 2.2. Fabrication and Setup of UFCT

Reagents: All reagents were purchased from commercial suppliers without further purification. Monodisperse fluorescent microspheres (polystyrene microspheres, ∼10 mg/mL) with a diameter of 2 μm were obtained from Aladdin Industrial Corporation (Shanghai, China). Monodisperse fluorescent microspheres (polystyrene microspheres, ∼10 mg/mL) with diameters of 5 μm and 10 μm were provided by the Baseline Chromatography Technology Development Center (Tianjin, China). SU-8 2025 photoresist was purchased from Suzhou KeYi Materials Microtech Co., Ltd. (Suzhou, China). Polydimethylsiloxane (PDMS, Sylgard 184) was sourced from Dow Chemical Company (Lake Jackson, TX, USA). The synthetic hydrogel used in this paper is 30 wt% solution of polyethylene glycol diacrylate with a molecular weight of 400 (Laysan Bio. Inc., Arab, AL, USA).

Manufacturing of Lamb Wave Resonators: The Lamb wave resonators used in this study were fabricated using standard MEMS processes. The substrate was a silicon wafer cleaned with piranha solution (a mixture of concentrated sulfuric acid and 30% hydrogen peroxide). A cavity was etched into the substrate using reactive ion etching, followed by chemical vapor deposition to fill it with phosphosilicate glass (PSG), which was then polished away using chemical mechanical polishing; the PSG within the cavity served as a sacrificial layer. Next, a molybdenum film (200 nm) was deposited on the sacrificial layer as the bottom electrode via magnetron sputtering, followed by deposition of an aluminum nitride layer (1.5 μm) and another molybdenum film (200 nm, top electrode) on the sacrificial layer using the same method. Since the deposited aluminum nitride layer covered the sacrificial layer, the former was etched using potassium hydroxide wet etching and plasma etching to expose the latter for subsequent processing. Finally, a gold film was deposited by physical vapor deposition, which was then lifted off to serve as electrical interconnections. The prepared sacrificial layer was etched using diluted hydrofluoric acid to release the resonant cavity, forming a cantilever structure.

Manufacturing of Micro-chamber: (1) Mixing PDMS base and curing agent (10:1) and casting in a mold; (2) Vacuum degassing to remove bubbles; (3) Curing at 80 °C for 1.5 h; (4) Peeling, measuring height, and punching with 2 mm and 1 mm punches for a 1 mm internal diameter; (5) Cleaning in ethanol and water, drying with nitrogen, and storing in a sterile Petri dish.

Experimental Platform Setup: The experimental platform was assembled by fixing the device onto an RF test board (EVB), connecting the electrodes to signal lines and ground lines using gold wires, and attaching a circular PDMS chamber with a diameter of 1 mm to the substrate as a sample container. The setup included essential equipment such as a signal generator (Agilent N2181B) for producing RF signals and providing input power to the device, a power amplifier (Qualwave QPAR1R53337) with a gain of 33 dBm operating in the 100–500 MHz frequency band, a power isolator (EPool QCIB-350–430-S) working in the 350–430 MHz band to prevent damage to the device from excessive power, and an SMA interface connecting the device to the isolator using standard 50 Ω RF transmission lines. The instruments were connected according to the circuit diagram. Additional equipment included a bright-field microscope equipped with a CCD camera for observing particle trajectories within the microfluidic chamber and a high-speed camera capturing images at 6000 fps to calculate and track particle motion states, thereby estimating particle velocities.

Cell Staining: We stained cells to assess cell aggregation, following these specific steps: (1) Prepare a 1 mmol/L stock solution of calcein and dilute it to 50 μmol/L using PBS buffer. (2) Add 10% of the volume of the cell culture medium with the calcein solution to the cell culture medium containing cells at a density of 1 × $10^{6}$ per mL. (3) Incubate the cells at 37 °C for 15–30 min. (4) Wash the cells thrice with PBS buffer to remove extracellular calcein dye, completing the preparation of the cell sample.

## 3. Results and Discussion

Particles subjected to a UFCT field primarily experience acoustic radiation force (ARF), acoustic streaming force (ASF), and centrifugal force (CF). The acoustofluidic dimensionless parameter κ (defined as $\kappa =\pi d_{p}/\lambda$) [40], representing the relative relationship between acoustic wave properties and particle size, is a key parameter for analyzing the influence of traveling surface acoustic waves on particle motion in an acoustofluidic field. When κ < 1, acoustic streaming dominates; when κ > 1, ARF prevails. Given f = 380 MHz, for $d_{p}$ = 2 μm and 10 μm, κ is approximately 1.6 and 8, respectively, both greater than 1. Consequently, for UFCT, the ARF is dominant compared to the ASF. Moreover, since ARF typically scales proportionally with particle volume while resistance scales with particle radius, the ARF is more pronounced for larger particles. Thus, the 10 μm particle experiences a stronger ARF than the 2 μm particle.

Additionally, CF is also an important force that cannot be overlooked, influencing the motion of particles within vortices. The vortex velocity is related to the amplitude of the acoustic wave, which determines the magnitude of the CF. It is well-known that the CF is directly proportional to the square of the angular velocity of the centrifugal motion ($CF\propto \omega^{2}$). When particles rotate at high speeds within the vortex, the CF increases. When this force becomes sufficient to overcome the acoustic vortex resistance, it begins to dominate the particle’s movement. The specific UFCT particle-capture process is described as follows: the LWR array couples into the fluid, generating first-order acoustic pressure effects and second-order acoustic streaming effects. The first-order acoustic pressure effect produces an ARF that pushes particles towards the center.

**Figure 3:**
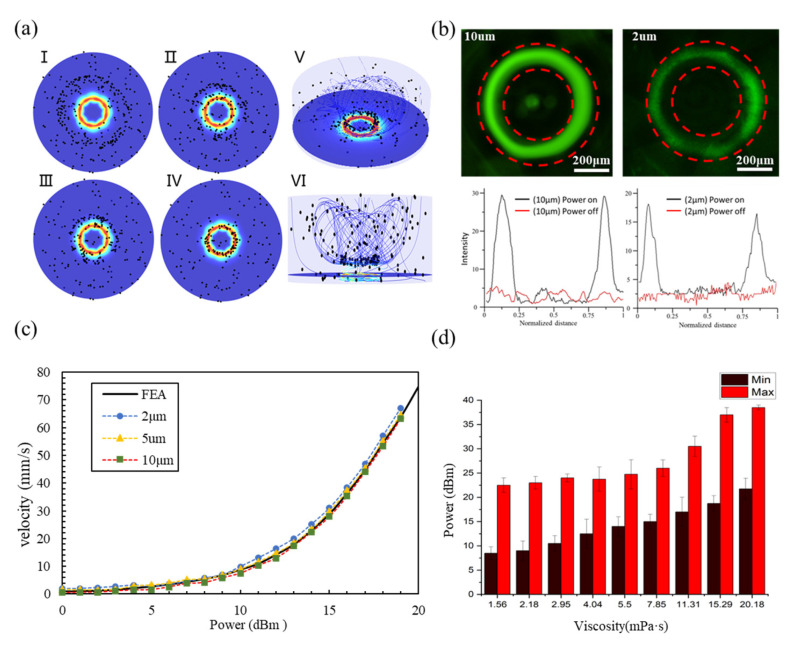
Particle simulation and experimental characterization based on UFCT (**a**) Simulation illustrating the particle capture process in UFCT. (**b**) Comparison of fluorescence intensity along the droplet axis before and after enrichment in UFCT, demonstrating the enrichment effect on different particles. (**c**) Graph depicting the relationship between the linear velocity of particles of various diameters and power. (**d**) Diagram showing the normal operating power range of UFCT under varied viscosities.

However, due to the presence of a single-sided reflector grating at ultra-high frequencies and the cooperation of a ring array, a larger portion of energy is rapidly converted, driving faster vortex motion. As a result, the CF acting on particles in the fluid rapidly increases and becomes dominant. Initially, particles move along streamlines towards the acoustic vortex center under the combined influence of CF and ARF. Upon reaching the virtual tunnel, the CF rapidly decreases. While the ARF pushes particles towards the center of the acoustic vortex, the enhanced CF acts in opposition, pushing particles away from the center. A stable particle ring forms where CF ≈ ARF, achieving an equilibrium state.As depicted in Figure 3a, for the simulation of particle enrichment by the UFCT, the chamber radius was set to 1.5 mm with a height of 1 mm. Subfigures (I–VI) illustrate the particle tracking simulation from initiation until the completion of enrichment, with time points at 0 s, 10 s, 20 s, and 30 s, respectively. Evidently, particles progressively accumulate from the edges towards the center. A dynamic visualization of this process can be found in the GIF S1. Figure 3a (V) and (VI) represent the oblique and side views of particle accumulation after enrichment. Particles are concentrated in a track approximately 300 μm above the device, a height that correlates with the attenuation distance of the acoustic waves generated by the device. Consequently, $F_{C}$ and ARF jointly govern the ring-like motion of particles around a specific acoustic vortex streamline. The results obtained are similar to those of previous reports [29,41], but the supplied power of UFCT is found to be an order of magnitude lower. For specific comparisons, please refer to Table S1. Subsequently, the enrichment effect of UFCT on particles of different sizes (2 μm and 10 μm) was characterized by measuring the fluorescence intensity before and after UFCT activation, as shown in Figure 3b.

For this purpose, the fluorescence intensity was statistically analyzed using ImageView software, with the fluorescence intensity values plotted on the vertical axis and the normalized distance on the horizontal axis. From the normalized curve, it is readily apparent that, prior to UFCT activation, particles are uniformly distributed within the cavity. Upon UFCT activation, however, enriched fluorescent particles predominantly accumulate, with widths of approximately 100 μm and radii around 250 μm. Notably, the 10 μm particles experience a stronger acoustic radiation force compared to the 2 μm particles, making them more prone to entering the central region of the virtual tunnel. Consequently, they exhibit higher fluorescence enhancement factors (seven-fold higher for 2 μm particles), consistent with previous studies [42].

To further characterize the velocity and behavior of particles, the number of rotations made by particles along the annular track was tallied, allowing for the calculation of their rotational speed. The linear velocities of polystyrene beads with diameters of 2 μm, 5 μm, and 10 μm were then computed under varying power levels, and these experimental data were compared with simulation results, as depicted in Figure 3c. It is evident that particles’ linear velocity increases rapidly with increasing power, and interestingly, under identical power settings, particles of different diameters exhibit nearly identical linear velocities. Moreover, these experimental findings closely align with the simulation results, thereby further substantiating the reliability of the experimental design. At a power level of 50 mW, a linear velocity of up to 62 mm/s was achieved.

To further investigate the adaptability of UFCT to real samples that typically possess varying viscosities, glycerol was added to the suspension medium to adjust its viscosity within the range of 1 to 20 mPa·s, as depicted in Figure 3d. This range includes the viscosities of all body fluids, such as cerebrospinal fluid (∼1 mPa·s), whole blood (4–5 mPa·s) [43], and low-molecular-weight hydrogels (more than 10 mPa·s). At lower power levels, the relatively weak fluid shear forces were insufficient to form the UFCT, whereas excessively high power could induce turbulence to destroy the UFCT. Thus, the higher the viscosity of the liquid, the greater the startup power needed to initiate particle movement along trajectories, and correspondingly, a higher power is required to displace particles from these trajectories. This also suggests that, in appropriate viscous environments, the dependence of particle or fluid motion on power decreases, implying an increased controllability of the particle dynamics. Moreover, it was demonstrated that UFCT can function effectively even in high-viscosity liquids, maintaining a wide operational power range. This finding lays a foundation for subsequent validation of cell assembly within hydrogels using UFCT technology.

**Figure 4:**
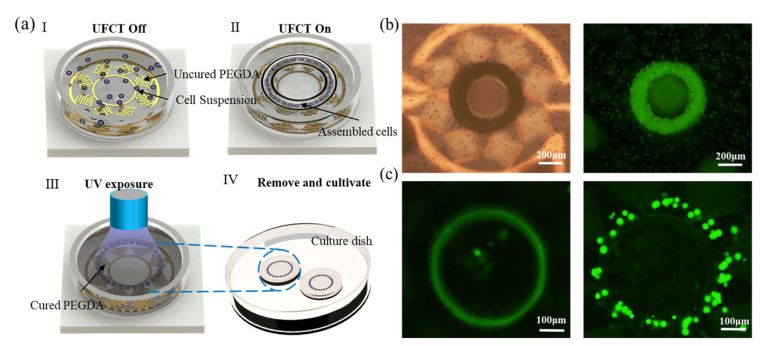
Cell enrichment and assembly based on UFCT. (**a**) Schematic representation of the cell enrichment assembly process facilitated by UFCT. (**b**) Comparative brightfield and fluorescence microscopy images of a 10 μm particle sample after UV curing. (**c**) The arrangement of cells before device activation and after UV exposure.

Tissue engineering and organoid technologies have greatly advanced our understanding of fundamental life science issues, including tissue development, intercellular interactions, and microenvironmental regulation. They provide researchers with unique platforms for ex vivo manipulation and observation of intricate biological processes [44,45,46]. This section validates the cell manipulation and assembly capabilities of UFCT. We conduct experiments on microtissue assembly using HeLa cells mixed with PEGDA, following the preparation steps (I)–(IV) depicted in Figure 4a: (I) Sample injection: mix the prepared cell sample with PEGDA at a ratio of 7:3, agitating it 10 times with a pipette to ensure homogeneity. Using a pipettor, aspirate 50 μL of the PEGDA/HeLa cell mixture and inject it into the microfluidic chamber atop the device. (Ⅱ) Enrichment assembly: turn on the signal generator and power amplifier, adjusting the power to 50 mW to generate UFCT and facilitate cell enrichment assembly. (Ⅲ) UV curing: Once cells have aggregated and formed ring-like structures, irradiate the structure for 15 s to photopolymerize the hydrogel structure. (Ⅳ) Removal and cultivation: Gently peel the cured structured micro-tissue off the PDMS wall and transfer it to a culture dish for further cultivation.

Figure 4b presents the manipulation results of a 10 µm particle after UV curing. Particles are employed to further validate the array’s excellent aggregation performance in high-viscosity PEGDA solutions, with images shown under both brightfield and fluorescence microscopy. Subsequently, we proceeded with HeLa cell assembly. Prior to power activation, cells are uniformly suspended in the solution. Upon power initiation, within tens of seconds, the majority of cells are captured and rapidly rotate within the central primary vortex, as shown in Figure 4c and Video S2. The process is also documented in Video S1. Ultimately, the assembly is UV-cured; excessively long exposure times render the PEGDA hard and brittle, compromising cell viability; an irradiation time of 10–20 s is deemed appropriate. The resulting cured structure, as seen in Figure 4c, faithfully preserves the ring-like configuration established during acoustic manipulation, with a radius of approximately 250 µm. This non-contact acoustic assembly approach offers a novel perspective for three-dimensional tissue engineering.

## 4. Conclusions

Acoustic microcentrifugation based on acoustic streaming has attracted significant attention due to its non-contact nature, high degree of miniaturization, and integration, particularly in the biochemical domain, where it serves applications ranging from driving micro-scale mixing to pre-concentration of samples and particles [47,48]. While maintaining compactness, achieving higher efficiency in the conversion of acoustic energy to acoustic streaming and stronger local fluid gradients are persistent objectives in acoustic microcentrifugation technology. This paper addresses these goals in two ways: first, by employing higher-frequency LWRs which enable rapid conversion of acoustic energy to streaming and can be driven at lower power levels, and secondly, through the implementation of a reflective grating and an arrayed configuration, concentrating more energy in the primary vortex while eliminating secondary vortices, thus maximizing the efficiency of converting acoustic waves to streaming energy. Furthermore, this paper explores preliminary applications of acoustic microcentrifugation in sample enrichment and cell assembly, successfully demonstrating the high-speed virtual tunnel created at low power and high-fold enrichment particles. An initial investigation into the potential use of UFCT in tissue engineering is also presented. This device holds promise for controlling the width of the vortex ring by adjusting input power and frequency, or tuning the diameter of the ring through the design of different array scales and device layout angles, enabling the creation of controllable microscale assemblies for studying cell–cell interactions and cellular responses to drug molecules in pharmaceutical development [49].

## Data Availability

The data that support the findings of this study are available from the corresponding author upon reasonable request.

## Supplementary Materials

The following supporting information can be downloaded at: https://www.mdpi.com/article/10.3390/bios14060280/s1, Figure S1. Comparison of flow field simulation with different numbers of LWR Arrays. Table S1. Comparison of different mechanisms and performance of acoustofluidic in concentrating particle ring. Video S1: Cell enrichment and assembly based on UFCT. Video S2: UFCT On. GIF S1: simulation via UFCT On.
