# Supplementary material for: Concentration of Microparticles/Cells Based on an Ultra-Fast Centrifuge Virtual Tunnel Driven by a Novel Lamb Wave Resonator Array
Source: Biosensors (Basel). 2024 May 29;14(6):280. doi: 10.3390/bios14060280 (PMC11202289; doi:10.3390/bios14060280)
Supplement: Supplementary file 1 [file biosensors-14-00280-s001.zip › biosensors-3002182-Support Information-for conversion.pdf]

biosensors

Supplementary Material

Concentration of Microparticles/Cells Based on an Ultra-Fast Centrifuge Virtual Tunnel Driven by a Novel Lamb Wave Resonator Array

Wei Wei, Zhaoxun Wang, Bingnan Wang, Wei Pang, Qingrui Yang and Xuexin Duan

1. The Design Concept of Different Numbers of LWR Arrays

The design of the LWR array boasts high flexibility, particularly with the assurance of impedance matching, enabling the number of resonators to be adjusted according to the requirements. This study primarily examines the design from two crucial perspectives: first, ensuring the flow field morphology, specifically maintaining a continuous annular virtual channel; second, optimizing device power consumption while ensuring that the flow field achieves maximum linear velocity and efficient particle enrichment without incurring additional energy expenditure. To preserve the symmetry of the flow field, employing an even number of devices becomes imperative. Consequently, simulations comparing configurations with 4, 6, 8, and 10 devices were conducted.

Under the condition that the device dimensions remain unchanged, each device's impact on the flow field is generally consistent. Arrays configured with 4 devices (illustrated in Figure S1 (a) and (b)) exhibit uneven flow velocity distributions due to the limited number of devices, which disrupts the continuity of the virtual channel and thereby hampers effective particle enrichment. Conversely, arrays consisting of six devices (Figure S1 (c)) display a more coherent flow pattern. Upon increasing to eight devices (as depicted in Fig. Ⅱ of 2(c)), the flow field successfully forms a continuous acoustic streaming virtual channel. Further augmentation to 10 devices (Figure S1 (d)) expands the virtual channel radius up to 600 μm, enhancing the throughput capacity. However, this does not tangibly increase the maximum linear velocity or fluid shear forces—which are critical factors for improving particle enrichment efficiency—while it does lead to increased power consumption.

Considering both the continuity of the flow field and the optimization of power consumption, the research concludes that an array composed of eight devices represents the most suitable configuration. This selection ensures a uniform flow field and an uninterrupted virtual channel, achieving effective particle enrichment without significantly escalating energy consumption.

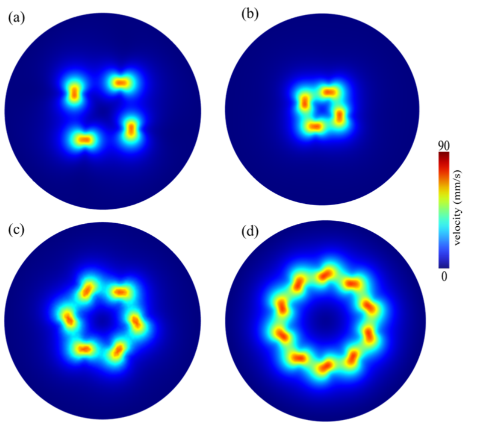

Figure S1. Comparison of flow field simulation with different numbers of LWR Arrays.

2. Specific Steps of COMSOL Simulation

The computational workload of the overall model is as follows. Initially, the piezoelectric effect of the Lamb wave resonator and the linear acoustic velocity field within the fluid domain are calculated using the solid mechanics, electrostatics, and pressure acoustics modules to determine the impedance-frequency relationship and the resonant frequency of the resonator. Subsequently, in the fluid domain of the established model, a laminar flow module is applied, accompanied by the introduction of a volumetric force. The velocity field solution is then utilized in the formula for the volumetric force, ultimately yielding the simulated acoustic streaming velocity field. For particle simulation, a particle simulation module is further incorporated, where particle trajectories are governed by drag forces from the fluid velocity field and acoustic radiation forces generated by the device.

In the context of a 2D simulation model, the steps are outlined as follows.

A 2D spatial modeling environment is chosen, with the inclusion of "Solid Mechanics," "Electrostatics," and "Pressure Acoustics" modules, each with their respective domains defined. The 2D geometry of the Lamb wave resonator is constructed, comprising a piezoelectric film, electrodes, and a fluid domain. The materials are assigned to the corresponding geometric regions: AlN for the piezoelectric layer, Mo for the electrodes, water for the fluid domain, and Si for the substrate. Loss factors are included, and boundary conditions are set. These encompass radiation boundaries and hard acoustic field boundaries for the fluid domain, along with acoustic–structure boundaries in the multiphysics context. Meshing is performed. Frequency-domain solving is conducted to obtain the displacement field and sound pressure field at the resonant frequency. Through finite element simulation, a 2D model of the novel Lamb wave resonator is established, with resonant frequencies of 380 MHz. In this resonator, the fluid domain near the resonator edges experiences a fluctuating pressure field, and the presence of air gratings on one side channels the acoustic wave leakage into the fluid unidirectionally, creating a volumetric force solely on this side. Moreover, the pressure intensity and gradient increase on the air grating side, enhancing the acoustic streaming effect there.

Owing to the geometric attributes of the resonant cavity, vibrations at its boundaries act as acoustic sources in the liquid and can be conceptualized as "line sources." Consequently, the acoustic waves in the vicinity of the lateral boundaries of the resonant cavity can be interpreted as cylindrical waves. The acoustic streaming phenomenon induced in the liquid by the Lamb wave resonator is precisely triggered by these cylindrical waves. Within the Lamb wave resonator and along its lateral boundaries, the displacement amplitude distribution of its fundamental mode vibration can be mathematically described by Equation 1, where W denotes the electrode stripe width, L signifies the electrode stripe length, and u_0 represents the amplitude of the displacement field.

$$u_s = u_0 cos\left(\frac{\pi}{W}x\right) sin\left(\frac{\pi}{L}y\right) \tag{1}$$

Furthermore, in accordance with the wave equation governing cylindrical waves, the velocity fluctuation equation close to the resonator's edge can be formulated as Equation 2, with β representing the attenuation coefficient of the sound wave, k being the wave number, and r symbolizing the propagation distance of the sound wave.

$$\boldsymbol{v}_1 = \frac{\boldsymbol{v}_{10}}{\sqrt{r}} e^{i(-kr+\omega t)} e^{-\beta r} \tag{2}$$

Upon extracting the first-order velocity field from the linear acoustic analysis, this information is incorporated into Equation 1 to calculate the volumetric force, which is subsequently introduced into the domain influenced by the volumetric force. The upper boundary of the fluid domain is designated as an "open boundary," effectively simulating an infinitely extended liquid medium. The computational results showcasing the steady-state laminar flow for the innovative Lamb wave resonator are illustrated in Fig. 2, with

the resonator's position outlined by a dashed black box. Due to the unidirectional characteristic of the volumetric force, the fluid migrates along the y-axis in line with the acoustic wave propagation, eventually circulating back towards the resonator's tail end, thereby generating vortices on both sides.

For particle trajectory simulations, the "Fluid Flow Particle Tracking" module is added, with particles possessing a density of 1050 kg/m³ and a diameter of 5 µm. Forces acting on particles—drag, gravity, and acoustic radiation forces—are included, with drag forces following the steady-state acoustic streaming velocity field, and initial particle velocities set to zero. The particle inlet and outlets are defined, with particles randomly released at the start. The computation is set to "Transient," solving over a period of 5 seconds with a time step of 0.001 seconds.

3. Comparison of Different Mechanisms and Performance of Acoustofluidic

Table S1. Comparison of different mechanisms and performance of acoustofluidic in concentrating particle ring.

Device	Mechanisms	Frequency	Throughput	Driving Power	Max linear speed	Size of particles	Other Applications
TSAW [41]	asymmetric propagation of travelling SAW	99.1MHz	8 µL	1000 mW	/	5-40µm	/
SSAW [42]	standing surface acoustic waves	98 MHz	5ul	160 mW	/	3µm	Separation of 7um and 3um PS particles
OS-SAW[29]	spiral surface acoustic waves	56.5 MHz	1.5 µl	Above 500 W	100 mm/s	5µm	Separation and extraction of red blood cells (RBCs) from mouse blood
UFCT	novel Lamb wave ring array	380 MHz	50ul	50mw	62 mm/s	2-10µm	The arrangement of cells for three-dimensional tissue engineering
