# Supplementary figures and images for: Concentration of Microparticles/Cells Based on an Ultra-Fast Centrifuge Virtual Tunnel Driven by a Novel Lamb Wave Resonator Array
Source: Biosensors (Basel). 2024 May 29;14(6):280. doi: 10.3390/bios14060280 (PMC11202289; doi:10.3390/bios14060280)

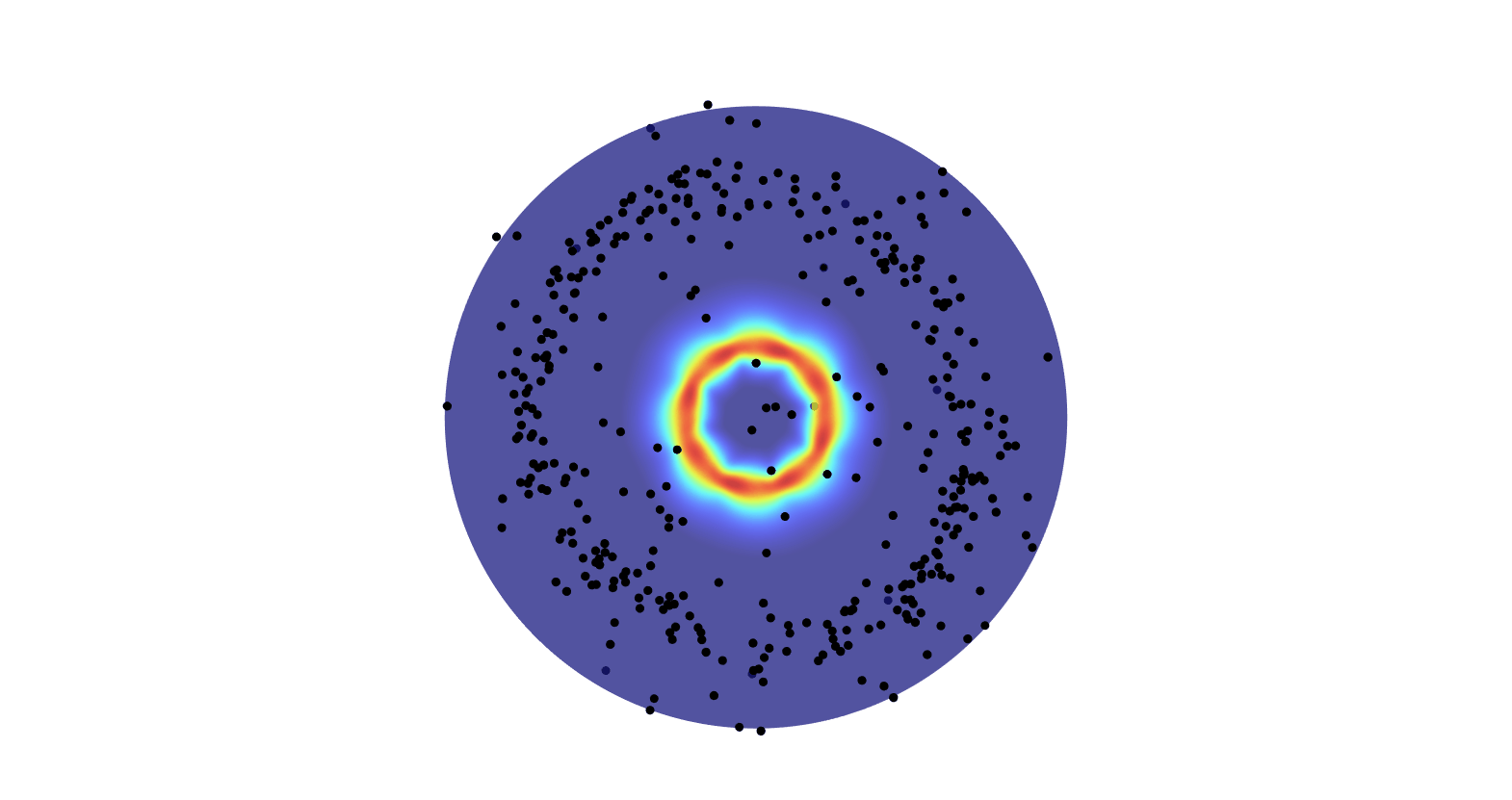

Supplement: Supplementary file 1 [file biosensors-14-00280-s001.zip › GIF 1. simulation via UFCT On.gif]
